# Gesättigte Fettsäuren und kardiovaskuläres Risiko: Ist eine Revision der Ernährungsempfehlungen angezeigt?

**DOI:** 10.1007/s00059-021-05067-6

**Published:** 2021-09-23

**Authors:** N. Worm, O. Weingärtner, C. Schulze, K. Lechner

**Affiliations:** 1 Deutsche Hochschule für Prävention und Gesundheitsmanagement, Saarbrücken, Deutschland; 2 Klinik für Innere Medizin I, Interventionelle Kardiologie/Angiologie, Universitätsklinikum Jena, Jena, Deutschland; 3 Klinik für Herz- und Kreislauferkrankungen, Deutsches Herzzentrum München, München, Deutschland

**Keywords:** LDL-Cholesterin, Kardiovaskuläre Erkrankungen, Prävention, Nährstoffe, Lebensstil, Milchfett, LDL cholesterol, Cardiovascular diseases, Prevention, Nutrients, Lifestyle, Dairy fat

## Abstract

Die „Fetthypothese der koronaren Herzkrankheit“, derzufolge „gesättigte Fettsäuren“ („saturated fatty acids“, SFA) die LDL(„low-density lipoprotein“)-Cholesterin-Konzentration (LDL-C) steigern und folglich das Risiko für kardiovaskuläre Erkrankungen erhöhen, prägte die Ernährungsempfehlungen der letzten 60 Jahre, zunächst in den USA und später auch in Europa. Über die Jahre mehrte sich Evidenz aus Epidemiologie und kontrollierten klinischen Studien, dass der Konsum von SFA per se nicht mit einem erhöhten kardiovaskulären Risiko einhergeht bzw. die Einschränkung des Konsums von SFA keine präventive Wirkung zeigt. Die Fokussierung auf den SFA-Gehalt negiert die biologisch heterogenen und zum Teil biologisch günstigen Wirkungen unterschiedlicher SFA. Zudem wird hierbei außer Acht gelassen, dass SFA in intakten Lebensmitteln in unterschiedliche komplexe Matrizes eingebunden sind, die aus Dutzenden Nährstoffen mit unterschiedlicher Struktur und Begleitstoffen bestehen und damit jeweils unterschiedliche biologische Antworten und metabolische Effekte auslösen. Entsprechend sind solche nährstoffbasierten Empfehlungen prinzipiell wenig zielführend und zudem schlecht umsetzbar. Hinzu kommt, dass LDL‑C kein geeigneter Marker ist, um den Effekt von Lebensstilintervention wie der Ernährung oder aber der körperlichen Aktivität auf das globale kardiovaskuläre Risiko zu beurteilen.

## Abstract (en)

The “fat hypothesis of coronary heart disease”, according to which saturated fatty acids (SFA) increase the concentration of low-density lipoprotein-cholesterol (LDL-C) and consequently increase the risk for cardiovascular diseases, influenced the nutritional recommendations over the last 60 years, initially in the USA and later also in Europe. Over the years there accumulated a growing body of evidence from epidemiology and controlled clinical studies that the consumption of SFA per se was not associated with an increased cardiovascular risk and the limitation of consumption of SFA did not show a preventive effect. The focus on the SFA content negated the biologically heterogeneous and sometimes biologically favorable effects of various SFAs. In addition, it was neglected that SFAs in foodstuffs are bound in a variety of complex matrices, which are composed of dozens of nutrients with different structures and concomitant substances and therefore each triggers different biological responses and metabolic effects. Accordingly, such nutrient-based recommendations are principally not very productive and also difficult to realize. In addition, LDL‑C is not a suitable marker to assess the effect of lifestyle interventions, such as nutrition or physical activity, on the global cardiovascular risk.

## Einführung

In den 1940er-Jahren wurden von John Gofman erstmalig Lipoproteine separiert und anhand der Dichte in verschiedene Klassen eingeteilt [1]. In einer Serie kontrollierter Stoffwechselexperimente beschrieb der Biochemiker Ancel Keys etwa zeitgleich, dass gesättigte Fettsäuren („saturated fatty acids“, SFA) die Serumcholesterinkonzentration (Gesamt-C) und die LDL(„low-density lipoprotein“)-Cholesterin-Konzentration (LDL-C) anheben, während einfach und mehrfach ungesättigte Fettsäuren („mono-unsaturated fatty acids“ [MUFA] bzw. „poly-unsaturated fatty acids“ [PUFA]) diese absenken [2]. Diese Ergebnisse konnten von Mark Hegsted repliziert werden [3]. In Zusammenschau mit der Beobachtung einer Assoziation zwischen erhöhtem Gesamt‑C bzw. LDL‑C und koronarer Herzkrankheit (KHK) in prospektiven Kohortenstudien (z. B. Framingham-Studie und Seven Countries Study; [1, 4–6]) wurde um 1960 die sog. Diet-heart-Hypothese (Fetthypothese der KHK bzw. „Fetthypothese“) formuliert.

Darauffolgende epidemiologische Erhebungen fielen uneinheitlich aus und kontrollierte Diätinterventionsstudien mit patientenrelevanten klinischen Endpunkten widersprachen in der Summe der „Fetthypothese“ (siehe Abschnitte „Prospektive Kohortenstudien“ und „Klinische Studien“; [7, 8]). Aufgrund einer steigenden Inzidenz der KHK und dem damit verbundenen Handlungsdruck wurden im Jahr 1977 von den US-amerikanischen Gesundheitsbehörden erstmals konkrete Ernährungsempfehlungen zur KHK-Prävention an die Bevölkerung abgegeben. Sie umfassten, der „Fetthypothese“ folgend, die Empfehlung einer Reduktion des Fettkonsums auf 30 Energieprozent (E%), des SFA-Anteils auf höchstens 10 E% und der Nahrungscholesterinzufuhr auf höchstens 300 mg/Tag [9]. Trotz fehlender bzw. schwacher Evidenzbasis und entsprechender Kritik [10–13] wurden im Jahre 1994 diese starken Handlungsempfehlungen von Fachgesellschaften in den USA (National Cholesterol Education Project der USA; [14]) sowie beispielsweise in England [15] und Deutschland [16] weitestgehend unverändert übernommen. Die zunehmende Diskrepanz zwischen Evidenz und Empfehlungen an die Bevölkerung [17–23] führte bis dato nicht zum Umdenken bei den Fachgesellschaften.

## Gesättigte Fettsäuren – eine biologisch heterogene Gruppe

Die Nomenklatur der Fettsäuren unterscheidet rein nach chemischer Struktur gesättigte, einfach ungesättigte und mehrfach ungesättigte Fettsäuren. SFA werden weiter in kurzkettige (2 bis 5 C-Atome), mittelkettige (6 bis 12 C-Atome), langkettige (13 bis 20 C-Atome) und sehr langkettige (21 bis 24 C-Atome), nichtverzweigte und verzweigte FS eingeteilt. Diese Einteilung lässt keine einheitlichen biologischen Effekte ableiten. So sind beispielsweise die Serumspiegel zweier im Milchfett vorkommender SFA mit einem niedrigeren kardiometabolischen Risiko assoziiert [24]. Hingegen ist für die gesättigte Palmitinsäure (C16:0) sowie für die durch ihre Desaturierung entstehende Palmitoleinsäure (C16:1 n‑7) und 7‑Hexadecensäure (C16:1 n‑9) eine positive Assoziation mit metabolischem Syndrom (MetS), Typ-2-Diabetes und kardiovaskulären Erkrankungen beschrieben [25–29]. Bemerkenswert und von zentraler Bedeutung für das Verständnis des Zusammenhangs zwischen SFA und kardiovaskulärem Risiko ist allerdings, dass diese bei Insulinresistenz und Hyperinsulinämie in hohem Maße aus Kohlenhydraten mittels De-novo-Lipogenese (DNL) von Myristinsäure (C14:0) und Palmitinsäure (C:16:0) entstehen [30] und entsprechend auch als Biomarker für die DNL dienen [31].

## Physiologische Wirkungen gesättigter Fettsäuren

Die verschiedenen SFA erfüllen im Organismus unterschiedliche biologische Aufgaben [32, 33]. Experimentell wirken bestimmte SFA potenziell risikosteigernd (siehe Abschnitt „Gesättigte Fettsäuren und Lipidstoffwechsel“), andere sind mit potenziell protektiven Effekten assoziiert (siehe Tab. 1; [32, 34–37]): Z. B. wird Buttersäure (C4:0) mit verstärkter Thermogenese, erhöhter Fettoxidation, verminderter hepatischer Fettsynthese, erhöhtem Energieverbrauch, verminderter Gewichtszunahme, gesteigerter Insulinsensitivität und verbessertem Zuckerstoffwechsel, Senkung von Blutlipiden sowie Minderung der Inflammationsneigung in Verbindung gebracht; für Valeriansäure (C5:0) sind antiinflammatorische und blutlipidsenkende Effekte nachweisbar; für Capronsäure (C6:0) finden sich antibakterielle und antivirale Effekte; für die Caprylsäure (C8:0) werden ebenfalls antivirale Aktivität, verminderte Sekretion von Apolipoprotein B (ApoB) und VLDL‑C („very low-density lipoprotein cholesterol“), verminderte DNL und verstärkte glukosestimulierte Insulinsekretion diskutiert; Caprinsäure (C10:0) greift in die hepatische Lipogenese ein, zeigt dabei eine cholesterin- wie auch triglyzeridsenkende Wirkung, mindert die intestinale Entzündungsneigung und den oxidativen Stress und stärkt die Darmbarriere; Laurinsäure (C12:0) stimuliert die GLP(„glucagon-like peptide“)-1-Ausschüttung und senkt damit die postprandiale Glukose und zeigt herzfrequenz- und blutdrucksenkende Wirkung; Pentadecansäure (C15:0) aus Milchfett wirkt in vivo antiinflammatorisch, antifibrotisch, stabilisiert Erythrozyten und aktiviert Reparaturmechanismen in Mitochondrien.

**Table 1:** 

Gesättigte Fettsäuren	Potenziell risikosteigernd	Potenziell risikomindernd
Buttersäure (C4:0)	–	Verstärkt Thermogenese, erhöht Fettoxidation, vermindert hepatische Fettsynthese, erhöht Energieverbrauch, vermindert Gewichtszunahme, steigert Insulinsensitivität, verbessert Zuckerstoffwechsel, senkt Blutlipide, mindert Inflammationsneigung
Valeriansäure (C5:0)	–	Antiinflammatorisch, blutlipidsenkend
Capronsäure (C6:0)	–	Antibakteriell und antiviral
Caprylsäure (C8:0)	–	Antiviral, vermindert Sekretion von ApoB und VLDL‑C, vermindert De-novo-Lipogenese, verstärkt glukosestimulierte Insulinsekretion
Caprinsäure (C10:0)	–	Mindert hepatische Lipogenese, senkt Blutlipide, mindert intestinale Entzündungsneigung und oxidativen Stress, stärkt die Darmbarriere
Laurinsäure (C12:0)^a^	Erhöht LDL‑C	Erhöht HDL‑C^a^, senkt Triglyzeride^a^, senkt Gesamt-C:HDL‑C^a^ und LDL-C:HDL‑C^a^, stimuliert GLP-1-Ausschüttung und senkt postprandiale Glukose, senkt Herzfrequenz- und Blutdruck
Myristinsäure (C14:0)^a^	Erhöht LDL‑C	Erhöht HDL‑C^a^, senkt Triglyzeride^a^
Pentadecansäure (C15:0)	–	Antiinflammatorisch, antifibrotisch, stabilisiert Erythrozyten, aktiviert Reparaturmechanismen in Mitochondrien
Palmitinsäure (C16:0)^a,b^	Erhöht LDL‑C, aktiviert Inflammasom	Erhöht HDL‑C^a^, senkt Triglyzeride^a^

*ApoB* Apolipoprotein B, *VLDL‑C* „very low-density lipoprotein cholesterol“, *HDL‑C* „high-density lipoprotein cholesterol“, *LDL‑C* „low-density lipoprotein cholesterol“, *GLP‑1* „glucagon-like peptide 1“

^a^im isokalorischen Austausch gegen Kohlenhydrate (nach [53])

^b^bei Insulinresistenz/Hyperinsulinämie in hohem Maße per De-novo-Lipogenese aus Stärke/Zucker synthetisiert

Für diese Effekte liegen mehrheitlich keine randomisierten, kontrollierten Interventionsstudien („randomized controlled trials“, RCT) zu klinischen Endpunkten vor. Allerdings deuten Beobachtungsstudien mit ihrer inversen Assoziation zwischen Markern des Milchfettkonsums (Serumspiegel von C15:0 und C17:0) und dem kardiometabolischen Risiko auf präventive Effekte hin (siehe Abschnitt „Wirkung gesättigter Fettsäuren in intakten Lebensmitteln“).

## Blutlipide und kardiovaskuläres Risiko – ein Paradigmenwechsel

Die kausale Rolle von ApoB-haltigen Lipoproteinen, also von VLDL, IDL („intermediate-density lipoprotein“, = „remnants“), LDL und Lipoprotein (a) (Lp[a]), bei der Atherogenese gilt als belegt. Allerdings weisen mehrere Linien der Evidenz darauf hin, dass LDL‑C, der klinisch gebräuchlichste Surrogatmarker für ApoB, keine verlässlichen Rückschlüsse auf die Beeinflussung des kardiovaskulären Risikos durch Ernährungsinterventionen zulässt [38–40]. So zeigten RCT mit mediterraner Kost eine deutliche Minderung kardiovaskulärer Endpunkte ohne signifikante Senkung des LDL‑C [41, 42]. Erwähnenswert in diesem Zusammenhang ist auch, dass eine Therapie mit SGLT2(„sodium-glucose linked transporter 2“)-Hemmern trotz einer Erhöhung des LDL‑C um 5–10 % [43] bei Patienten mit Typ-2-Diabetes, Herzinsuffizienz und chronischer Nierenerkrankung zu einer signifikanten Reduktion kardiovaskulärer Endpunkte führte [44–46]. Andere Medikamente wie Hormonersatzpräparate (Östrogen/Progestin) oder auch eine Klasse der Lipidsenker (Cholesterinesterproteininhibitoren) erzielten wiederum trotz deutlicher Senkung des LDL‑C keine Reduktion kardiovaskulärer Endpunkte [40].

Eine höhere Vorhersagekraft für das kardiovaskuläre Risiko als LDL‑C hat das Non-HDL(„high-density lipoprotein“)-C (Gesamtcholesterin minus HDL-C), da es die Cholesterinkonzentration aller atherogen wirkenden Lipoproteine abbildet. V. a. bei Menschen mit Insulinresistenz und MetS ist aufgrund qualitativer Veränderungen im Lipidprofil im Sinne einer Zunahme von VLDL‑C dieser Parameter zur Risikobeurteilung besser geeignet als LDL‑C [47]. Noch genauer ist die Vorhersagekraft von ApoB [48] als direktem Maß für die Partikelanzahl aller atherogenen Partikel [39, 40, 49, 50]. Denn es ist zu beachten, dass es in bestimmten metabolischen Situationen – beispielsweise bei nichtalkoholischer Fettlebererkrankung („non alcoholic fatty liver disease“, NAFLD), MetS und Diabetes mellitus – zur erheblichen Diskordanz von LDL‑C und ApoB (Marker für die LDL-Partikel-Anzahl) kommen kann. Dies erklärt sich durch Veränderungen des LDL-Partikel-Phänotyps bei erhöhtem hepatischen Triglyzeridpool. Dadurch kommt es im Rahmen einer Reihe von koordinierten pathophysiologischen Veränderungen im Lipidprofil zur Bildung großer, triglyzeridreicher VLDL-Partikel, woraus wiederum kleine und cholesterindepletierte LDL-Partikel entstehen. In dieser Situation ist die LDL-C-Konzentration im Vergleich zur LDL-Partikel-Anzahl (ApoB) diskordant niedrig und führt bei dieser Subgruppe von Patienten (≤ 50 % der Bevölkerung) zur erheblichen Unterschätzung des atherogenen Risikos [39, 51]. Dies begründet auch die Empfehlung der ESC(European Society of Cardiology)/EAS(European Atherosclerosis Society)-Leitlinie „Dyslipidämie“, bei Patienten mit Diabetes mellitus, hohen Triglyzeriden und mit niedrigem LDL‑C die Messung von ApoB und Non-HDL zur genaueren Risikostratifizierung eine Messung von ApoB dem LDL‑C vorzuziehen (Empfehlungsklasse 1, Evidenzgrad A; [47]). Kurz zusammengefasst, bildet LDL‑C das Risiko adäquat ab, solange es mit ApoB, d. h. mit der Partikelanzahl, konkordant ist. Zu beachten ist, dass dies bei einer substanziellen Subgruppe der Bevölkerung jedoch nicht der Fall ist, z. B. bei Patienten mit NAFLD, MetS und Diabetes mellitus [52].

Die atherogene Dyslipidämie, die pathognomonische Fettstoffwechselstörung bei MetS und gestörter Glukosetoleranz/Typ-2-Diabetes, kann in der Regel im Routinelabor erkannt und durch eine LDL-Partikel-Differenzierung genauer phänotypisiert werden. Im Routinelabor hinweisend sind erhöhte Triglyzeride, ein erniedrigtes HDL‑C sowie ein meist normales LDL‑C. Das erweiterte Lipoproteinprofil zeigt eine relative Zunahme triglyzerid- und ApoC3-angereicherter VLDL-Partikel (VLDL1) sowie einen erhöhten Anteil an kleinen LDL-Partikeln (LDL III und LDL IV), einen erhöhten Anteil an kleinen HDL-Partikeln und ein meist erhöhtes ApoB [51]. Pathophysiologisch zurückzuführen sind diese koordinierten Veränderungen des Lipoproteinphänotyps auf einen erhöhten hepatischen Triglyzeridpool [51]. Die NAFLD, inzwischen als Risikofaktor für kardiovaskuläre Erkrankungen anerkannt [53], ist hierbei das Bindeglied. Bemerkenswert ist, dass der pathophysiologisch aus dem erhöhten hepatischen Triglyzeridanteil resultierende Lipidphänotyp klinisch mit dem Vorliegen eines TCFA(„thin-cap fibroatheroma“)-Plaque-Phänotyps assoziiert ist [39, 40, 50]. Dieser Zusammenhang könnte über die verlängerte Verweildauer im Plasma und die damit erhöhte Expositionszeit des arteriellen Endothels mit proinflammatorischen und proatherogenen Bestandteilen, wie ApoC3, und der damit in der Summe erhöhten Atherogenität begründet sein [39, 40, 49, 50].

## Gesättigte Fettsäuren und Lipidstoffwechsel

3 SFA (C12:0, C14:0 und C16:0) gehen in Stoffwechselexperimenten mit einem Anstieg von LDL‑C einher [54]. Bei der gemeinhin empfohlenen Minderung der SFA-Zufuhr – im isokalorischen Austausch gegen Kohlenhydrate – kommt es zur Senkung von Gesamt‑C, LDL‑C und ApoB, aber auch von ApoA und HDL‑C, und zur Steigerung der Triglyzeridkonzentration. Die Verhältnisse Triglyzeride:HDL‑C, Gesamt-C:HDL‑C bzw. LDL-C:HDL‑C werden nicht signifikant verändert [54]. Beim Austausch von SFA gegen PUFA oder MUFA wie Linolsäure (C18:2 n‑6) oder Alpha-Linolensäure (C18:3 n‑3) oder Ölsäure (C18:1n‑9) kommt es zu einer Senkung von ApoB, Gesamt‑C und LDL‑C, allerdings auch von und ApoA und HDL‑C. Gesenkt werden auch Triglyzeride, Triglyzeride:HDL‑C und Gesamt-C/HDL‑C [54].

Relevanter sind vermutlich die qualitativen Veränderungen des Lipoproteinprofils, welche in einem Standardlipidprofil nicht ersichtlich sind. So kommt es durch Senkung der SFA-Zufuhr in der Regel zu einer Minderung der Anteile großer LDL-Partikel [40, 49]. Meist erreicht man damit eine Senkung des Surrogatmarkers LDL‑C, nicht aber eine nennenswerte Minderung von ApoB. Damit könnte eine diätetisch erzielte Senkung des LDL‑C die präventive Wirkung deutlich überschätzen [40]. Formeln zur Risikovorhersage, die primär LDL‑C fokussieren, lassen nach einer Beschränkung der gegenwärtig durchschnittlichen SFA-Zufuhr von 12–15 E% auf 5 E% eine Senkung des LDL‑C um etwa 0,25 mmol/l (9,7 mg/dl) erwarten. Das wiederum resultiert theoretisch in einer Senkung des kardiovaskulären Risikos um etwa 5 % [40]. Bei den oben aufgezählten ungünstigen Einflüssen einer SFA-Senkung auf ApoA, HDL, LDL-Partikel-Größe und triglyzeridreiche Remnant-Partikel würde dies aber möglicherweise eine präventive Wirkung der LDL-Senkung weitgehend oder gänzlich kompensieren [40].

### Infobox 1 Kurzgefasst: Gesättigte Fettsäuren und Lipoproteine jenseits der LDL-Konzentration

Im Austausch gegen Kohlenhydrate bewirken SFA konzeptionell eine Zunahme der LDL-Partikel-Größe (einhergehend mit Anstieg des LDL‑C, aber keinem oder nur geringem Effekt auf ApoB), einen Anstieg von ApoA bzw. HDL‑C und eine Reduktion des Triglyzeridanteils in VLDL-Partikeln und Chylomikronen-Remnants. Ein Austausch von MUFA und PUFA gegen SFA zeigt konzeptionell einen Anstieg von ApoB und ApoA bzw. LDL‑C und HDL‑C sowie einen neutralen Effekt auf Gesamt-C:HDL‑C und Triglyzeride [54, 55].

## Phänotyp und Lipidstoffwechsel

Generell gilt, dass der Einfluss von SFA auf den Lipidstoffwechsel entscheidend vom Kohlenhydratanteil der Nahrung, von der Kalorienbilanz und der Insulinsensitivität abhängig ist. So steigt unter kohlenhydratbetonter, fettarmer Kost der Anteil an kleinen LDL-Partikeln, die, wie auch postprandiale Remnant-Partikel, in großen Kohortenstudien mit erhöhtem kardiovaskulären Risiko assoziiert waren. Dieser Lipidphänotyp wird gefördert durch eine langfristige positive Kalorienbilanz (durch Überernährung und/oder sedentären Lebensstil), insbesondere in Zusammenschau weiterer Faktoren wie Schlafmangel, Dysstress etc., was wiederum die Entstehung von Insulinresistenz fördert. Mit der dadurch bedingten Hyperinsulinämie werden Kohlenhydrate über die DNL zu hohen Anteilen in Fett umgewandelt und in der Leber eingelagert. Die Steatose fördert die Ausprägung der atherogenen Dyslipidämie unabhängig vom Körpergewicht [56]. Beim dyslipidämischen, metabolischen Phänotyp mit erhöhtem hepatischen Triglyzeridpool entstehen triglyzeridreiche VLDL (VLDL1), die besonders reich an der durch DNL synthetisierten Palmitinsäure (C16:0) sind [39, 57]. Durch Anreicherung mit Apolipoprotein C3 und dem charakteristischen Partikellipidom (z. B. Palmitinsäure C16:0) wirken der VLDL- und der LDL-Phänotyp bei Insulinresistenz und NAFLD proinflammatorisch und bilden somit eine gewisse Brücke zwischen Lipid- und inflammatorischem Risiko [58].

Umgekehrt nimmt bei deutlicher Kohlenhydratreduktion und hoher Fettzufuhr („low carb“) – selbst bei hohem Anteil von SFA – die Produktion von größeren LDL-Partikeln zu und der Anteil triglyzeridreicher Lipoproteine ab [40]. Diese insbesondere bei der Subgruppe von Patienten mit Insulinresistenz, MetS, NAFLD, gestörter Glukosetoleranz und Typ-2-Diabetes günstig einzuschätzenden Effekte sind auf Veränderungen der Körperzusammensetzung zurückzuführen, v. a. auf die Abnahme ektoper Fettdepots [59, 60]. So kommt es bei strikter Kohlenhydratrestriktion zum Abbau von Leberfett, da hierbei selbst ohne Energiereduktion die hepatische Fettoxidation gesteigert und die DNL gemindert ist [51, 61, 62].

## Genetische Varianten und Lipidstoffwechsel

### Gesättigte Fettsäuren

Wenngleich die Effektstärken gering sind, ist eine weitere Erklärung für die Verschiebung im Lipoproteinprofil durch SFA die genetische Prädisposition [33]. So ist für Varianten im ApoE-Gen (*APOE*) eine ungünstige metabolische Lipoproteinantwort auf alimentäre SFA beschrieben [51]. Konkret konnte gezeigt werden, dass Träger der weniger häufigen *APOE4*-Allele nach einer definierten Menge von SFA [63] und postprandial [64] einen ausgeprägteren Lipidanstieg im Plasma aufweisen, als Nicht-*APOE4*-Träger [33]. Diese Gen-Diät-Interaktionen (Nutrigenetik) zeigten sich auch für Adipositas. So interagierte der Konsum von SFA mit einem gewichteten genetischen Risikoscore für das Adipositasrisiko [64]. Dabei fand sich die Assoziation zwischen SFA-Aufnahme und Body-Mass-Index (BMI) nur in der oberen Terzile, d. h. es sprechen jene 33 % der Bevölkerung mit einer stärkeren genetischen Disposition zur Adipositas sensitiver auf SFA an [64].

Diese Daten weisen darauf hin, dass es genetische Unterschiede bei der Metabolisierung von SFA gibt. Ein regelmäßiges Monitoring von Biomarkern ist somit bei Ernährungsinterventionen wie auch bei pharmakologischen Interventionen anzuraten, solange prädiktive Biomarker für Therapieansprechen nicht verfügbar sind [65].

### Sterole

Als Folge der empfohlenen Meidung von SFA wurden ab den 70er-Jahren vermehrt pflanzliche Öle und Streichfette mit hohen Anteilen an ungesättigten Fettsäuren „zur Senkung des Cholesterinspiegels“ empfohlen. Eine vermehrte Aufnahme von Phytosterinen (2 g/Tag) über solche sog. Functional Foods – wie beispielsweise Margarine – senkt LDL‑C um bis zu 10 %. Dies hat zur Empfehlung von Phytosterinen zur LDL-C-Senkung in der aktuellen ESC/EAS-Leitlinie zum Management der Dyslipidämien geführt [47], der das Paradigma zugrunde liegt, dass jede LDL-C-senkende Lebensstilmaßnahme empfehlenswert sei. In den letzten Jahrzehnten kamen noch „funktionelle Nahrungsmittel“ hinzu, die mit Phytosterinen angereichert sind, um über deren Cholesterinresorption hemmende Wirkung das LDL‑C noch ausgeprägter zu senken [47].

Der Serumcholesterinspiegel wird einerseits durch die endogene Cholesterinbiosynthese (hepatische und extrahepatische Cholesterinbiosynthese) und andererseits durch die intestinale Cholesterinresorption reguliert [66]. Die Cholesterinhomöostase wird somit durch ein Gleichgewicht zwischen Cholesterinresorption und Cholesterinsynthese erreicht: Menschen mit hoher intestinaler Cholesterinresorption weisen eine niedrige endogene Cholesterinsynthese auf, während Menschen mit niedriger intestinaler Cholesterinresorption durch eine hohe endogene Cholesterinsyntheseleistung gekennzeichnet sind [67]. Lathosterol, eine Vorstufe der endogenen Cholesterinbiosynthese, kann als Marker für die Cholesterinsynthesekapazität bestimmt werden [68]. Diese individuellen Unterschiede in der Cholesterinhomöostase sind genetisch reguliert und haben einen starken Einfluss auf die Effektivität LDL-C-senkender Medikamente, was sich auch in einer unterschiedlichen Reduktionsrate von kardiovaskulären Ereignissen widerspiegelt. So profitieren Menschen mit hoher intestinaler Cholesterinresorption deutlich weniger von einer Statintherapie [69], während Menschen mit hoher intestinaler Cholesterinresorption und damit erhöhten Phytosterinkonzentrationen besonders von einer Hemmung der Cholesterinresorption durch Ezetimib profitieren [70].

Nur in pflanzlichen Organismen werden Phytosterine (Campesterin und Sitosterine) synthestisiert. Sie unterscheiden sich strukturchemisch von Cholesterin lediglich durch eine Methyl- bzw. Ethylgruppe. Deren Resorption dient zur Bestimmung der intestinalen Cholesterinresorptionskapazität [68]. Cholesterin und Phytosterine liegen ungefähr zu gleichen Anteilen in der westlichen Ernährung vor (400 mg/Tag). Beide werden in Mizellen transportiert und über NPC1-L1 („Niemann-Pick C1-like 1“) in den Enterozyten aufgenommen. Im Enterozyten (und später auch im Hepatozyten) werden sie aber voneinander unterschieden. Während das intestinal resorbierte Cholesterin bis zu 50 % verestert und im weiteren Verlauf dem Stoffwechsel zur Verfügung steht, werden die Phytosterine „erkannt“ und über ABCG5/8 („ATP-binding cassette sub-family G member 5/8“) in das Darmlumen zurückgepumpt. Dieser Mechanismus funktioniert beim „gesunden“ Menschen so effektiv, dass 98 % der Phytosterine wieder intestinal ausgeschieden werden und die Phytosterinkonzentration im Blut etwa 1000-fach geringer ist als die des Cholesterins [71].

Bei der sehr seltenen autosomal-rezessiv vererbten Erkrankung Sitosterolämie ist aufgrund eines Defekts von ABCG5/8 die Phytosterinausscheidung kompromittiert. Es kommt zu einem bis zu 100-fachen Anstieg der Phytosterinkonzentrationen im Plasma [72]. Diese Menschen sind gekennzeichnet durch eine sehr frühe maligne Atherosklerose, hochgradige Aortenstenosen und oft durch einen frühzeitigen kardiovaskulären Tod. Das Verständnis dieser Erkrankung sowie weitere epidemiologische Untersuchungen haben den Verdacht genähert, das Phytosterine *per se* atherogen sind. Diese Hypothese wurde in einer kürzlich publizierten genetischen Analyse an knapp 100.000 Menschen verifiziert. Helgadottir et al. untersuchten den Einfluss aller in den Cholesterin- und Phytosterinstoffwechsel eingebundenen Gene auf das kardiovaskuläre Risiko [73]. In ihrer Analyse unterschieden sie Gene, die Cholesterin, aber nicht Phytosterine beeinflussen, wie beispielsweise ApoB, LDL-Rezeptor, HMG-CoA(3-Hydroxy-3-Methylglutaryl-Coenzym-A)-Reduktase und Proproteinkonvertase Subtilisin/Kexin Typ 9 (PCSK9), von Genen, die Cholesterin und Phytosterine beeinflussen wie ABCG5, ABCG8 und NPC1L1. Für jede Erhöhung des Non-HDL‑C um 1 mmol/l steigerten Gene, die nur Cholesterin beeinflussen, das kardiovaskuläre Risiko um den Faktor 1,5. Gene, die neben dem Serumcholesterinspiegel auch den Serumphytosterinspiegel durch Minder- respektive Überexpression des Steroltransporters ABCG5 (und NPC1L1), beeinflussen, erhöhten das Risiko jedoch 2‑fach.

Zusammenfassend ist zu sagen, dass der sehr seltene komplette Funktionsverlust des Proteins ABCG5/8 (= Sitosterolämie) von in der Prävalenz unterschätzten genetischen Varianten abzugrenzen ist, welche zwar eine geringer ausgeprägte, aber klinisch relevante Sterolaufnahmestörung verursachen [73], und es über die vermehrte Zufuhr von Phytosterinen auch zu einer Verdopplung der Phytosterinkonzentrationen im Plasma kommen kann [74]. Insbesondere Menschen mit einer genetischen Konstellation von ABCG5/8 und NPC1L1, die zu einer vermehrten Sterolaufnahme führt, sind hierbei besonders gefährdet [67]. Vor dem Hintergrund der Atherogenität ist die Empfehlung von Phytosterinen zur LDL-C-Senkung in der aktuellen ESC/EAS-Leitline zum Management der Dyslipidämien [47] kritisch zu sehen, und möglicherweise wird sich eine Änderung dieser Empfehlung ergeben [71, 73].

Umgekehrt könnten Patienten mit Varianten in den Genen, die für die apikalen Steroltransportproteine ABCG5/G8 kodieren, von einer Reduktion von diätetischen Sterole und von einer pharmakologischen Therapie mit Ezetimib besonders profitieren.

Diese Erkenntnis hat weitreichende Konsequenzen, da sie verständlich macht, dass die Betrachtung von LDL‑C für die Einschätzung des kardiovaskulären Risikos und für personalisierte Therapieentscheidungen nicht ausreichend ist. Die genauere gaschromatographische Bestimmung der individuellen Unterschiede im Hinblick auf die Cholesterinhomöostase von Lathosterin (Marker für endogene Cholesterinsynthese) wie auch von Campesterin und Sitosterin (als Marker für Cholesterinresorption) ermöglicht eine differenziertere Betrachtung und Identifizierung von Menschen mit einer hohen Sterolresorption. Diese „high resorber“ sollten frühzeitig diätetisch betreut und einer individualisierten lipidsenkenden Therapie zugeführt werden [67].

## Gesättigte Fettsäuren und kardiovaskuläre Erkrankungen

Die isolierte Betrachtung des Einflusses einzelner Fettsäuren auf ausgewählte Surrogatparameter kardiovaskulärer Gesundheit wie z. B. das LDL‑C ermöglicht keine sinnvolle klinische Einschätzung des Effekts von Lebensmitteln oder Ernährungsmustern auf das globale kardiovaskuläre Risiko und ist daher wohl wenig zielführend.

### Prospektive Kohortenstudien

In den letzten 40 Jahren wurden zahlreiche prospektive Kohortenstudien zum Einfluss der SFA auf kardiovaskuläre Erkrankungen durchgeführt. Mehrheitlich hatten sie gezeigt, dass der SFA-Konsum kein unabhängiges kardiovaskuläres Risiko darstellt, d. h. weder mit kardiovaskulärer Sterblichkeit noch mit Gesamtsterblichkeit assoziiert ist [7, 17, 75]. Metaanalysen haben die Studien zusammenhängend gewichtet und bewertet [76–82] und die fehlende Assoziation zwischen SFA-Konsum und KHK bestätigt. Auch zwischen der Blutkonzentration an SFA und KHK konnte kein Zusammenhang beobachtet werden [79], was auch bei einer deutschen Kohorte in der LURIC(Ludwigshafen Risk and Cardiovascular Health)-Studie bestätigt wurde [83]. Interessanterweise waren in mehreren Kohortenstudien erhöhte Plasmaspiegel der beiden Fettsäuren, die primär endogen durch DNL aus Kohlenhydraten entstehen (C16:0 und C16:1 n7), mit kardiovaskulärem Risiko und Mortalität assoziiert [84]. Demgegenüber fand man für die Serumspiegel der Fettsäuren, die als Marker für Milchfettkonsum dienen (C15:0 und C17:0), eine inverse Assoziation mit dem kardiometabolischen Risiko [37]. Dies spiegelt die Diskordanz zwischen alimentären und im Serum gemessenen SFA-Spiegeln wider [85].

In Beobachtungsstudien findet sich ein konsistenter inverser Zusammenhang zwischen SFA-Konsum und Schlaganfall [78, 86]. Auch die PURE(Prospective Urban Rural Epidemiology)-Studie mit Ernährungsdaten von 136.384 Teilnehmern aus 21 Ländern mit niedrigem, mittlerem und höherem Pro-Kopf-Einkommen und einem Follow-up von 7 Jahren bestätigte die Assoziation zwischen höherem SFA-Konsum und dem geminderten Schlaganfallrisiko (und auch der Gesamtsterblichkeit). Hingegen fand sie zwischen SFA-Konsum und Myokardinfarkt oder insgesamt bezüglich der kardiovaskulären Sterblichkeit keinen Zusammenhang [87].

Bedacht werden muss allerdings, dass Beobachtungs- bzw. Kohortenstudien insbesondere in der Ernährungsmedizin sehr störanfällig sind (unzuverlässige subjektive Erhebungsmethoden, Confounding durch unzureichende Adjustierung von relevanten Lebensstilfaktoren, „selection bias“ oder „residual confounding“, „healthy user bias“ etc.; [88–91]). Versuche, aus solchen Studien mittels rechnerischer Austauschmodelle (SFA gegen Kohlenhydrate oder gegen MUFA bzw. PUFA) eine Risikobeeinflussung darzustellen [92, 93], sind wenig pragmatisch und nicht beweisführend für kausale Zusammenhänge.

### Klinische Studien

Bei zahlreichen RCT wurde die „Fetthypothese“ bzw. der Effekt einer SFA-Senkung getestet, wobei SFA entweder durch Kohlenhydrate (fettreduzierte Diät) oder ungesättigte Fettsäuren (fettmodifizierte Diät) ausgetauscht wurden. Allerdings waren weitere RCT von einer unifaktoriellen Intervention abgewichen (Finnish Mental Hospital Study [FMHS], Oslo Diet-Heart Study [ODHS], St. Thomas’ Atherosclerosis Regression Study [STARS], Women’s Health Initiative Randomized Controlled Dietary Modification Trial [WHI]) und hatten erweiterte Ernährungsmodifikationen mit präventivem Potenzial (vermehrter Konsum von Früchten und Gemüsen/Nüssen/Fisch bzw. Einsatz von Margarinen mit Omega-3-Fettsäuren und Vitamin-D-Anreicherung/unterschiedliche Transfettsäurenzufuhr/Ballaststoffsupplementation/Minderung der Zuckerzufuhr/Gewichtsreduktion etc.) und damit multifaktorielle Interventionen getestet [75, 77, 94]. Zudem handelt es sich bei der FMHS nicht um eine RCT. In frühen Metaanalysen wurden dennoch alle als „Überprüfung der Fetthypothese“ zusammengefasst [75, 77, 94–105]. Mehrheitlich war dabei weder eine Reduktion der kardiovaskulären Mortalität noch der Gesamtsterblichkeit nachweisbar [106].

Ein im August 2020 aktualisierter Cochrane-Review schloss 15 RCT ein [107], darunter auch 3 mit multifaktorieller Intervention (ODHS, STARS, WHI). Die erzielte SFA-Senkung ergab im Vergleich zur Kontrollgruppe

- keine signifikante Senkung der Gesamtsterblichkeit (relatives Risiko [RR]: 0,96; 95 %-Konfidenzintervall [KI]: 0,90–1,03);
- keine signifikante Senkung der kardiovaskulären Sterblichkeit (RR: 0,95; 95 %-KI: 0,80–1,12);
- keine signifikante Senkung des nichttödlichen Myokardinfarkts (RR: 0,97; 95 %-KI: 0,87–1,07);
- keine signifikante Senkung der KHK-Sterblichkeit (RR: 0,97; 95 %-KI: 0,82–1,16);
- unklare Effekte auf Gesamtmyokardinfarkte (tödliche und nichttödliche), Hirninfarkte, KHK-Ereignisse (tödliche und nichttödliche);
- signifikante Senkung der Kombination von weichen und harten Endpunkten („kombinierte kardiovaskuläre Ereignisse“; RR: 0,83; 95 %-KI: 0,70–0,98);
- keine signifikante Senkung des Risikos (RR: 0,86; 95 %-KI: 0,67–1,09) zum kombinierten Endpunkt („kombinierte kardiovaskuläre Ereignisse“), bei einer Subanalyse nach Ausschluss von RCT mit multifaktorieller Intervention (ODHS, STARS, WHI).

## Wirkung gesättigter Fettsäuren in intakten Lebensmitteln

Etablierte Ernährungsempfehlungen kategorisieren SFA als biologisch einheitlich wirkende Gruppe und beurteilen die gesundheitliche Bedeutung von Lebensmitteln pauschal nach ihrem SFA-Gehalt, so auch der neu eingeführte Nutri-Score. Dies lässt ungeachtet, dass Menschen nicht isolierte Fettsäuren, sondern Lebensmittel mit unterschiedlichen SFA essen, die eine unterschiedliche Absorptionskinetik aufweisen, unterschiedlich transportiert und verstoffwechselt werden und unterschiedliche biologische Wirkungen auslösen. Zudem sind SFA in unterschiedliche, komplexe Matrizes eingebunden, die aus Dutzenden Nährstoffen mit unterschiedlicher Struktur und Begleitstoffen bestehen. Damit lösen unterschiedliche SFA-haltige Lebensmittel verschiedene biologische Antworten und metabolische Effekte aus, die weniger auf isolierten Fettsäuren als auf der Summe aller Bestandteile des Lebensmittels beruhen [108]. Das Milchfett beispielsweise, das tierische Fett mit dem höchsten SFA-Anteil, wird je nach Darreichungsform von Hunderten biologisch wirksamen Nähr- und Inhaltsstoffen begleitet, für die potenzielle gesundheitliche Vorteile bekannt sind [108]. Die verschiedenen Milchprodukte wirken in Abhängigkeit von Fermentations‑, Pateurisierungs- oder Homogenisierungsverfahren unterschiedlich auf den Lipidstoffwechsel, was gemeinhin in Ernährungsempfehlungen unberücksichtigt bleibt [109]. Seit Langem weist auch die Datenlage für vermehrten Konsum von Milch und Milchprodukten – auch von vollfetten – kein erhöhtes kardiovaskuläres und zum Teil sogar ein signifikant vermindertes Risiko aus [110–119]. Zudem sind die Marker für den Verzehr von Milchprodukten (C15:0 bzw. Pentadecansäure und C17:0 bzw. Heptadecansäure) invers mit dem Risiko für kardiometabolische Erkrankungen assoziiert [24, 37].

Für den Konsum von unverarbeitetem roten Fleisch, dessen Fettanteil im Gegensatz zur weit verbreiteten Meinung zu mehr als 50 % aus ungesättigten Fettsäuren besteht, konnte in kontrollierten Stoffwechselstudien bis dato kein Risikosignal für eine Erhöhung kardiometabolischer Risikofaktoren gezeigt werden [120]. Kohortenstudien und randomisierte, kontrollierte Interventionsstudien zu klinisch relevanten Endpunkten ergeben nach dem GRADE(Grading of Recommendations, Assessment, Development and Evaluation)*-*System nur „niedrige“ bis „sehr niedrige“ Evidenz für eine Senkung des kardiovaskulären Risikos durch Minderkonsum [121, 122]. Entsprechend ist ein klinisch relevanter Nutzen einer Einschränkung des Konsums von rotem Fleisch fraglich, und konkrete Empfehlungen zu einem Minderkonsum aus ernährungsphysiologischer Sicht sind möglicherweise wenig sinnvoll [123]. In dieser Hinsicht sollte erwähnt werden, dass es aus ethisch-moralischen wie auch aus ökologischen Gründen wohl sinnvoll ist, Fleisch aus artgerechter Haltung zu beziehen und den Konsum auf ein sinnvolles Maß zu beschränken.

**Figure 1:**
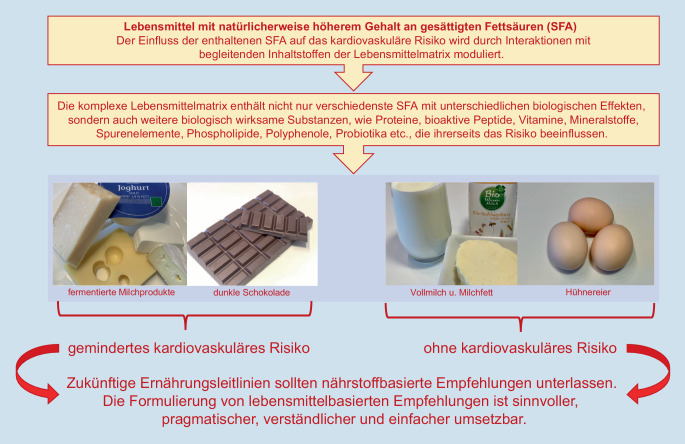

Zusammenfassend bewertet, zeigen verschiedene Linien der Evidenz, dass die Zufuhr von SFA – eingebunden in die natürliche Nahrungsmatrix z. B. bei naturbelassenen Milchprodukten, unverarbeitetem Fleisch, Eiern und Kakaoprodukten (Schokolade) – nicht das Risiko für kardiovaskuläre Erkrankungen und Sterblichkeit steigert (Abb. 1) [33].

## Schlussfolgerung und Perspektive

Insgesamt deutet die Evidenz nicht darauf hin, dass eine Reduktion SFA-haltiger Lebensmittel harte kardiovaskuläre Endpunkte oder die Sterblichkeit reduziert [33]. Entsprechend erscheint die Empfehlung vieler Leitlinien, wie auch der ESC/EAS-Leitlinie „Dyslipidämie“, Lebensmittel nach ihrem Gehalt an SFA zu bewerten und pauschal deren Minderverzehr zu empfehlen, wenig sinnvoll.

Die Effekte von Lebensmitteln auf das globale kardiovaskuläre Risiko resultieren einerseits aus der Wirkung der Summe der Bestandteile eines Lebensmittels und andererseits aus der Gesamtheit der Ernährung, welche wohl am verlässlichsten durch Ernährungsmuster abgebildet wird. Lebensmittelbasierte Empfehlungen wären deswegen nicht nur pragmatischer, sondern wohl auch sinnvoller als die isolierte Betrachtung einzelner Nahrungsbestandteile [33, 55, 65, 108, 124–130]. Die aktuell vorliegende Evidenz spricht hierbei für die Empfehlung von Essmustern reich an naturbelassenen, unverarbeiteten Nahrungsmitteln aus pflanzlichen und tierischen Quellen sowie arm an Zucker und raffinierter Stärke und frei von Transfettsäuren [50].

Für die metabolischen Bedürfnisse der immer größer werdenden Subgruppe der Bevölkerung mit Adipositas, Bewegungsmangel, Insulinresistenz und deren Folgen (MetS, NAFLD, Prä- und Typ-2-Diabetes) zeigen konsistente Daten aus mehreren Evidenzlinien einen günstigeren Einfluss für kohlenhydratreduzierte, ballaststoffreiche, fett- und proteinreichere Ernährungsformen auf Risikomarker als fettreduzierte Ernährungsmuster [39, 50, 131, 132].

Bei diesem dysmetabolischen, dyslipidämischen Phänotyp wirken sich insbesondere mediterran ausgerichtete, kohlenhydratreduzierte Essmuster günstig auf das kardiometabolische Risikoprofil und die Reduktion ektoper Fettdepots aus [50].

Genetische und erworbene Faktoren bestimmen individuell unterschiedliche metabolische Antworten auf Nahrungsmittel. Die Zukunft der Ernährungsempfehlungen liegt in der Integration und Übersetzung von Information über Genotyp (Nutrigenomics; [133]), metabolischen Phänotyp [39] und Umweltfaktoren [50] in personalisierte Empfehlungen. Die Anwendung künstlicher Intelligenz, Ableitungen aus tragbaren digitalen Messinstrumenten (Blutzuckermessung, Schlaftracking, Bewegungsmonitore etc.) und die weitere Erforschung des Metaboloms werden dazu beitragen, dem Individuum personalisierte Ernährungsentscheidungen an die Hand zu geben [133].
