# Erratum zu: Gesättigte Fettsäuren und kardiovaskuläres Risiko: Ist eine Revision der Ernährungsempfehlungen angezeigt?

**DOI:** 10.1007/s00059-021-05083-6

**Published:** 2021-11-25

**Authors:** N. Worm, O. Weingärtner, C. Schulze, K. Lechner

**Affiliations:** 1 Deutsche Hochschule für Prävention und Gesundheitsmanagement, Saarbrücken, Deutschland; 2 Klinik für Innere Medizin I, Interventionelle Kardiologie/Angiologie, Universitätsklinikum Jena, Jena, Deutschland; 3 Klinik für Herz- und Kreislauferkrankungen, Deutsches Herzzentrum München, München, Deutschland

**Erratum zu:**

**Herz 2021**

10.1007/s00059-021-05067-6

Im Abschnitt „Gesättigte Fettsäuren und Lipidstoffwechsel“ ist die Kettenlänge der Ölsäure falsch angegeben. Diese beträgt 18, nicht 16 Kohlenstoffatome. Der entsprechende Satz muss richtig lauten: „Beim Austausch von SFA gegen PUFA oder MUFA wie Linolsäure (C18:2 n‑6) oder Alpha-Linolensäure (C18:3 n‑3) oder Ölsäure (C18:1 n‑9) kommt es zu einer Senkung von ApoB, Gesamt‑C und LDL‑C, allerdings auch von und ApoA und HDL‑C.“

Die Autoren entschuldigen sich für den Fehler.

